# Plant-Based Antioxidant Extracts and Compounds in the Management of Oral Cancer

**DOI:** 10.3390/antiox10091358

**Published:** 2021-08-26

**Authors:** Suraj Prakash, Radha, Manoj Kumar, Neeraj Kumari, Mamta Thakur, Sonia Rathour, Ashok Pundir, Abhishek Kumar Sharma, Sneh Punia Bangar, Sangram Dhumal, Surinder Singh, Anitha Thiyagarajan, Anshu Sharma, Munisha Sharma, Sushil Changan, Minnu Sasi, Marisennayya Senapathy, Prakash Chandra Pradhan, Nitin Kumar Garg, Tamilselvan Ilakiya, Mukesh Nitin, Mohamed M. Abdel-Daim, Sunil Puri, Suman Natta, Abhijit Dey, Ryszard Amarowicz, Mohamed Mekhemar

**Affiliations:** 1 School of Biological and Environmental Sciences, Shoolini University of Biotechnology and Management Sciences, Solan 173229, India; surajpandiar75@gmail.com (S.P.); neeruguleria1532001@gmail.com (N.K.); mamtaparmar369@gmail.com (M.T.); soniagunnu1981@gmail.com (S.R.); sunilpuri@shooliniuniversity.com (S.P.); 2 Chemical and Biochemical Processing Division, ICAR—Central Institute for Research on Cotton Technology, Mumbai 400019, India; 3 School of Mechanical and Civil Engineering, Shoolini University of Biotechnology and Management Sciences, Solan 173229, India; ashok.pundir78791@gmail.com; 4 School of Pharmaceutical Sciences, Shoolini University of Biotechnology and Management Sciences, Solan 173229, India; Abhishek_sharma31@outlook.com; 5 Department of Food, Nutrition, & Packaging Sciences, Clemson University, Clemson, SC 29634, USA; snehpunia69@gmail.com; 6 Division of Horticulture, RCSM College of Agriculture, Kolhapur 416004, India; sdhumal@msu.edu; 7 Dr. S.S. Bhatnagar University Institute of Chemical Engineering and Technology, Panjab University, Chandigarh 160014, India; ssbhinder@pu.ac.in; 8 Department of Postharvest Technology, Horticultural College and Research Institute, Periyakulam 625604, India; anitha.anitha303@gmail.com; 9 Department of Food Science and Technology, Dr. Y.S. Parmar University of Horticulture and Forestry, Nauni 173230, India; anshufst1989@gmail.com; 10 Sri Shankara Cancer Hospital and Research Centre, Bengaluru 560004, India; munishamans@gmail.com; 11 Division of Crop Physiology, Biochemistry and Post-Harvest Technology, ICAR-Central Potato Research Institute, Shimla 171001, India; sushil.changan@icar.gov.in; 12 Division of Biochemistry, ICAR—Indian Agricultural Research Institute, New Delhi 110012, India; minnusasi1991@gmail.com; 13 Department of Rural Development and Agricultural Extension, College of Agriculture, Wolaita Sodo University, Wolaita Sodo, SNNPR, Ethiopia; drsenapathy@wsu.edu.et; 14 Division of Agricultural Chemicals, ICAR—Indian Agricultural Research Institute, New Delhi 110012, India; prakash0844@gmail.com; 15 Division of Biochemistry, Sri Karan Narendra Agriculture University, Jobner 303329, India; nkgarg.biochem.rari@sknau.ac.in; 16 Department of Vegetable Science, Tamil Nadu Agricultural University, Coimbatore 641003, India; ilakiyatamil@gmail.com; 17 Department of Tech. Biosciences, Digianalix, South Samaj Street, Tharpakhna, Ranchi 834001, India; digianalix@gmail.com; 18 Pharmacy Program, Department of Phamaceutical Sciences, Batterjee Medical College, P.O. Box 6231, Jeddah 21442, Saudi Arabia; abdeldaim.m@vet.suez.edu.eg; 19 Pharmacology Department, Faculty of Veterinary Medicine, Suez Canal University, Ismailia 41522, Egypt; 20 ICAR—National Research Centre for Orchids, Pakyong 737106, India; nattabiochem@gmail.com; 21 Department of Life Sciences, Presidency University, 86/1 College Street, Kolkata 700073, India; abhijit.dbs@presiuniv.ac.in; 22 Institute of Animal Reproduction and Food Research, Polish Academy of Sciences, 10-748 Olsztyn, Poland; amaro@pan.olsztyn.pl; 23 Clinic for Conservative Dentistry and Periodontology, School of Dental Medicine, Christian-Albrecht’s University, 24105 Kiel, Germany

**Keywords:** oral cancer, phytoconstituents, medicinal plants, oral squamous cell carcinoma

## Abstract

Oral cancer continues to be a leading cause of death worldwide, and its prevalence is particularly high in developing countries, where people chew tobacco and betel nut on a regular basis. Radiation-, chemo-, targeted-, immuno-, and hormone-based therapies along with surgery are commonly used as part of a treatment plan. However, these treatments frequently result in various unwanted short- to long-term side effects. As a result, there is an urgent need to develop treatment options for oral cancer that have little or no adverse effects. Numerous bioactive compounds derived from various plants have recently attracted attention as therapeutic options for cancer treatment. Antioxidants found in medicinal plants, such as vitamins E, C, and A, reduce damage to the mucosa by neutralizing free radicals found in various oral mucosal lesions. Phytochemicals found in medicinal plants have the potential to modulate cellular signalling pathways that alter the cellular defence mechanisms to protect normal cells from reactive oxygen species (ROS) and induce apoptosis in cancer cells. This review aims to provide a comprehensive overview of various medicinal plants and phytoconstituents that have shown the potential to be used as oral cancer therapeutics.

## 1. Introduction

According to Globocan 2020, oral cancer is a common malignancy, with 177,757 deaths and 377,713 new cases reported each year worldwide. Oral cavity cancer is very common in South-Central Asia (e.g., India, Pakistan, and Sri Lanka), with more than one-third of the new cases (135,929) and one-fifth of the deaths (75,290) occurring in India alone [1]. The most common oral cancer is oral squamous cell carcinoma (OSCC), which has a wide range of clinical manifestations and accounts for more than 90% of all oral cancers [2]. The major risk factors for oral cancer include chronic inflammation, human papillomavirus or *Candida* infections, alcohol and tobacco use, ultraviolet radiation, immunosuppression, genetic susceptibility, and diet. Despite the availability of novel therapeutic options, the 5-year survival rate for oral cancer in most countries remains below 50%. Tobacco and alcohol usage are two of the most significant risk factors for oral cancer [3]. Oral inflammation may also have a role in the pathophysiology of oral cancers due to the involvement of several inflammatory pathways, such as cyclooxygenase (COX)-2, phosphatidylinositol 3-kinase (PI3K/Akt), mitogen-activated protein kinases (MAPK), nuclear factor-κB (NF-κB), Janus kinase/signal transducer and activator of transcription (JAK/STAT) [4]. It has been reported that oral cancer patients have significantly higher levels of *Candida albicans* genotype A strains than noncancer patients, and these strains have also been linked to leukoplakic lesions [5]. Immunosuppression has also been linked to the growth of oral cancer in patients undergoing bone marrow transplantation and renal transplantation [6].

Despite several advancements in treatment options for oral cancer, the survival rates have not increased considerably over the last few decades. Therefore, new and effective treatments to prevent the development of oral cancer are required. In recent years, several authors have reported the potential of natural products and phytochemicals derived from plants in the treatment of oral diseases [7,8,9,10,11]. Several natural compounds have been investigated for their ability to cause apoptosis in human cancer cells [12]. These phytocompounds may cause cell death by inducing apoptosis and arresting the cell cycle and could be exploited for the treatment of oral cancer with less systemic toxicity and side effects in humans [13]. Traditional medicines are used by a significant proportion of the global population to treat a variety of diseases [14,15,16,17,18,19,20,21]. Curcumin, lycopene, ginseng, anthocyanins, and artemisinin are some of the potential compounds that have shown promising results against OSCC and other tumours.

To the best of our knowledge, a comprehensive review that considers the role of antioxidant plant extracts and their constituents in treating oral cancer has not been reported in the literature. Thus, this review provides information on medicinal plants and phytochemicals derived from them for the treatment of oral cancer and assesses studies that have examined their anticancer properties in vitro and in vivo.

## 2. Methodology

In this study, the anticancer properties of medicinal plants and their phytoextracts in oral cancer treatment were reviewed using the “Preferred Reporting Items for Systematic Reviews and Meta-Analyses” (PRISMA) 2020 guidelines [22]. Studies were selected based on the following inclusion criteria: (i) both endemic and cosmopolitan species were included based on less-frequently reviewed literature; (ii) original studies showing chemopreventive properties against oral cancer were selected; (iii) studies available in full text articles in English were included; and (iv) *in vitro*, in vivo and clinical trial studies showing authentic data were included (studies published in high-ranking journals were preferred).

The exclusion criteria were as follows: (i) Studies published in local languages (except English) were excluded; (ii) studies not having full text available were excluded; (iii) studies examining other types of cancer, e.g., lung cancer, colon cancer, etc., were excluded; and (iv) in vivo and in vitro studies not following ethical guidelines were excluded.

An electronic literature search was carried out in the following databases: Scopus, PubMed, Google Scholar, and Elsevier using the following keywords alone or in combination: oral cancer, carcinoma, types of oral cancer, medicinal plants, phytoextracts, anticancer agents, apoptosis, cell cycle arrest, and oral squamous cell carcinoma in vivo and *in vitro*. The literature search was performed from 24 March 2021 to 12 July 2021. Studies within the period from 2001 to 2021 were included in the review. A total of 157 studies were found from database searches, 23 duplicate records were removed, 14 studies with no full text were removed, and a total of 94 were selected for systematic review.

The name of medicinal plants was followed according to the plant list database [23]. After the selection of studies, the following data were collected: distribution of plants, source of phytoextract, anticancer properties of medicinal plants and phytoextracts, concentration of extract, the results of *in vivo*, in vitro and clinical trial studies.

**Figure 1:**
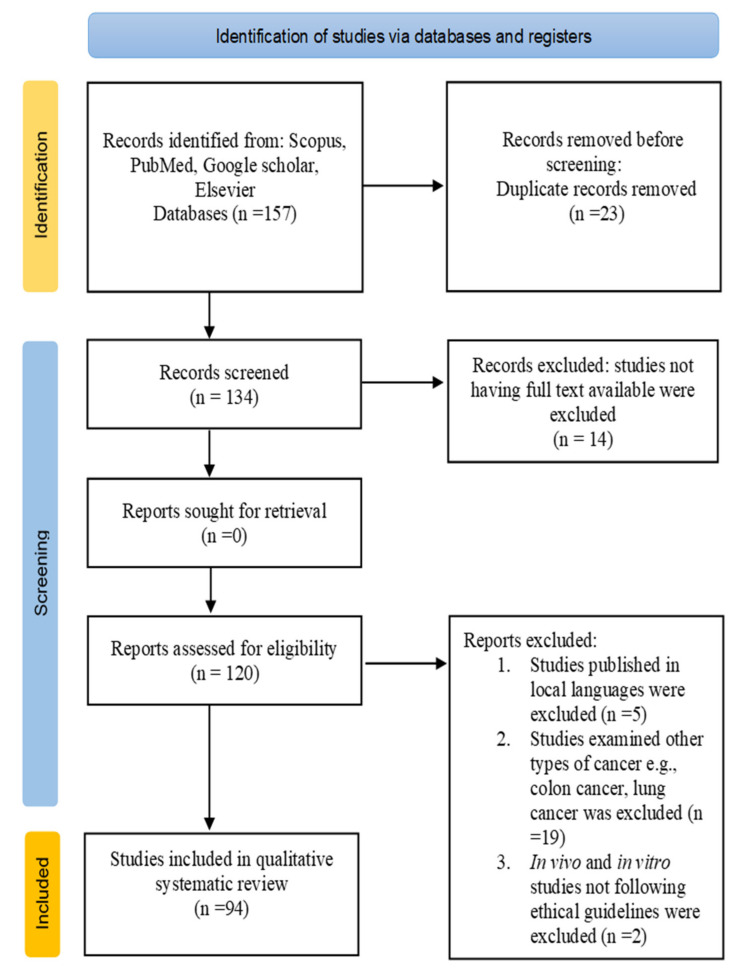
PRISMA flow diagram for the selection process of studies included in the current review.

The PRISMA flow diagram (Figure 1) shows the number of records identified, selection process, inclusion and exclusion criteria, and number of studies selected for the literature review.

## 3. Types of Oral Cancer

### 3.1. Oral Squamous Cell Carcinoma (OSCC)

OSCC dominates with an 84–97% occurrence rate among all oral cancer cases worldwide and is the 10th most common malignancy in females and the 6th most common malignancy in males. Approximately 50% of OSCC tumours report that a dysfunctional p53 gene plays an important role in checkpoint controls and apoptosis mechanisms. Potentially malignant disorders (PMDs), such as candidal leukoplakia, lichen planus, erythroplakia, inflammation, and dyskeratosis congenita, were indicators in the preclinical phase of oral cancer. OSCC commonly develops on the tongue, lips, and mouth surface [24]. The main therapeutic strategy in the treatment of OSCC includes a combination of radiation therapy and surgery. During treatment, patients were reported to have negative side effects in terms of oral function and appearance. In recent years, for OSCC treatment, many researchers have studied drugs obtained from medicinal plants that have fewer side effects and a better survival rate [25,26].

### 3.2. Oral Verrucous Carcinoma (VC)

Oral VC is a rare subtype of oral squamous cell carcinoma with cytodynamic features and specific morphologies. Oral VC accounts for 2% to 16% of all oral malignancies [27]. In early 1948, Lauren V. Ackermann first described oral VC, which is also referred to as “Ackermann’s tumour” [28]. Common oral cavity VC sites include the gingiva, mandibular alveolar crest, buccal mucosa, and tongue, and a common nonoral site is the glottic larynx. Drinking alcohol, the use of tobacco in inhaled and smokeless forms, and invariably poor oral hygiene are shown to be major causes in affected patients [29]. Oral VC is a low-grade malignancy that rarely metastasizes and has slow growth and a high degree of differentiation. Its proliferative and invasive outgrowing nature might induce the destruction of adjacent tissue, such as bone and cartilage. Numerous therapeutic protocols, including chemotherapy, lasers, and radiotherapy, have been employed in the treatment of oral VC (28).

### 3.3. Oral Melanoma

Oral melanoma, a metastatic cancer, is an aggressive lethal skin cancer and extremely rare (0.2–8%) among all malignant melanomas [30]. Melanoma is a type of cancerous tumour that results from the uncontrolled proliferation of pigment-producing cells called melanocytes [31]. The oral and nasal cavities are locations with the best prognosis. The first case of oral melanoma was identified in 1885 [32]. This type of cancer frequently affects males and occurs mostly in different sites of the oral cavity, such as the maxillary alveolar mucosa, gingiva, and hard palate. As the third most common malignancy, malignant melanoma represents only 3 to 5% of all cutaneous malignancies. The diagnosis for patients with oral melanoma is much worse than that for patients with cutaneous lesions, and the 5-year survival rate is approximately 15–38%. Oral malignant melanoma occurs on oral sites, including the floor of the mouth, lips, tongue and buccal mucosa. The palate is reported to be the most common site at 40% of cases, followed by the buccal gingiva, with one-third of cases [30].

### 3.4. Lymphoma

Lymphoma is a heterogeneous malignant tumour of the lymphatic system that results from the proliferation of lymphoid cells and their precursors. According to the alterations in their behavioural patterns and histology, they were divided into two groups: non-Hodgkin’s and Hodgkin’s lymphoma. Hodgkin’s lymphoma arises mostly in lymph nodes (>90%) [33]. Compared to squamous cell carcinoma, oral cavity lymphoma signifies the 3rd most commonly occurring malignancy in the oral cavity, and 4% of all patients with AIDS (acquired immune deficiency syndrome) suffer from lymphomas. The oral manifestations of lymphomas are difficult to diagnose because of their resemblance to the clinical features of other diseases, such as osteomyelitis, periodontal disease, and other malignancies [34]. Primary sites of oral manifestations of lymphomas in the oral cavity include the gingiva, floor of the mouth, palate, tongue, cheek and lips. Diagnosis is based on a combination of blood tests, selective biopsies, physical examination, and diagnostic imaging [33].

Considering the beneficial role of plant extracts and their phytoconstituents in oral cancer, the next Section 4 and Section 5 will discuss the role of plants and their constituents in the management of oral cancer.

## 4. Plants with Beneficial Effects against Oral Cancer

### 4.1. Ocimum sanctum *L*.

Ocimum sanctum, which is known as Holy Basil or Tulsi, is a sacred herb belonging to the Labiatae family, and it is richly cultivated worldwide. O. sanctum is reported to have numerous medicinal properties, such as anticaries, antifungal, anticancer, antibacterial and antiviral properties. Holy basil contains compounds such as flavonoids, alkaloids, ursolic acid, tannins, carbohydrates, and eugenol [35]. A study reported that O. sanctum has a cytotoxic effect against the KB oral cancer mouth cell line (mouth epidermal carcinoma cells), with IC_50_ values of 10 µg/mL (light leaf aqueous extract) and 20 µg/mL (dark leaf aqueous extract) [36]. In a recent study, O. sanctum leaf ethanolic extract was examined for its antiinvasive effect on head and neck squamous cell carcinoma (HNSCC) cell lines (HN4, HN12, HN30, and HN31). In the cytotoxicity assay, O. sanctum ethanolic extract (0.8 mg/mL) treatment showed a decrease in the viability of HNSCC cell lines HN30 (40%), HN31 (53%), HN4 (52%), and HN12 (40%) compared to the controls (*p* < 0.05). O. sanctum ethanolic extract at a concentration of 0.4 mg/mL inhibited matrix metalloproteinase (MMP)-2 activity in HN12 and HN4 cells by 71% and 65%, respectively, and MMP-9 activity in HN12 and HN4 cells by 85% and 44%, respectively. The invasion activity of HN12 and HN4 cells was inhibited by 30% in comparison with that of the control (*p* < 0.05), but no significant changes were observed in HN31 and HN30 cells [37]. In a recent study, the anticancer activity of O. sanctum was examined in the OSCC cell line Ca9–22. In a treatment with O. sanctum aqueous extract, the highest permissive concentration (HPC) value was 30 mg/L and minimum inhibitory concentration (MIC) was 5 mg/L, and in a treatment with the dry extract, the HPC value was 35 mg/L and MIC value was 5 mg/L. A neutral red uptake (NRU) assay showed that the lethal concentration (LC) values for the dry extract were 30.19 mg/L (LC_75_), 23.44 mg/L (LC_50_), and 16.59 mg/L (LC_25_) and those for the aqueous extract were 20.89 mg/L (LC_75_), 14.79 mg/L (LC_50_), and 10.23 (LC_25_). In a 3-(4,5-dimethythiazol-2-yl)-2,5-diphenyl tetrazolium bromide (MTT) assay, the LC values for the dry extract were 29.51 mg/L (LC_75_), 20.89 mg/L (LC_50_), and 12.58 mg/L (LC_25_) and those for the aqueous extract were 26.91 mg/L (LC_75_), 14.79 mg/L (LC_50_), and 7.41 mg/L (LC_25_) [35]. In an in vivo study, the anticancer activity of vicenin-2 (a bioactive compound found in O. sanctum) was examined in DMBA (7,12-dimethylbenz [a] anthracene)-induced OSCC hamsters. With the DMBA treatment, 100% tumour incidence was observed and cytokine levels (tumour necrosis factor-alpha [TNF-α], interleukin [IL]-1β, IL-6) were upregulated. Vicenin-2 treatment (30 mg/kg) with DMBA-induced OSCC hamsters improved antioxidant levels, inhibited lipid peroxidation, and halted tumour incidence. DMBA-induced hamsters treated with vicenin-2 showed a lack of production of proinflammatory cytokine (TNF-α, IL-1β, and IL-6) and inhibited immunohistochemical expression of cyclin D1, B-cell lymphoma 2 (Bcl-2), and proliferating cell nuclear antigen (PCNA). Vicenin-2 treatment activates apoptotic Bax expression, which further inhibits the expression of antiapoptotic Bcl-2 [38].

### 4.2. Curcuma longa *L*.

*Curcuma longa* (Turmeric), a medicinal herb belonging to the Zingiberaceae family, is native to Southeast Asia. Turmeric is known for its medicinal properties, such as antimicrobial, anti-inflammatory, hepatoprotective, antiseptic, and antimutagenic properties, and it is used in the treatment of oral cancer and periodontal diseases [39]. Turmeric contains chemical compounds known as curcuminoids, including bisdemethoxycurcumin-curcumin, curcumin, and dimethoxy-curcumin. A recent study reported that curcumin is the chief component of turmeric and has an antitumour effect in HNSCC [40]. In a recent study, the antitumour effect of curcumin (with copper supplementation) treatment with OSCC cell Lines H314 and ORL-115 obtained from cancer patients was examined. In the presence of 250 µM copper, a decrease in curcumin concentrations was observed, which inhibited 50% of cell viability in OSCC (IC_50_), 25 μM to 5.3 μM at 48 h and 50 μM to 40.3 μM at 24 h. With an increase in copper levels in OSCC cells treated with curcumin, an increase in Nrf2 levels and significant induction of intracellular ROS were observed. Curcumin along with copper treatment at 24 h compromised cell membrane integrity as a late event in apoptosis, and this treatment at 6 h induced apoptotic DNA fragmentation [41]. In another in vitro study, the effect of curcumin on epithelial-mesenchymal transition (EMT) induced by hepatocyte growth factor (HGF) in the OSCC cell lines Ca9–22 and HSC4 was observed. The results showed that curcumin treatment in OSCC cells inhibited cell motility and HGF-induced EMT via c-Met blockade. Curcumin treatment reduced phosphorylated c-mesenchymal epithelial transition/extracellular signal-regulated kinase (Met/ERK) pathway expression, which inhibited the HGF-induced increase in vimentin levels [42].

### 4.3. Vaccinium corymbosum *L.*

*Vaccinium corymbosum* is commonly known as blueberry, belongs to the Ericaceae family and is distributed among different countries, such as Canada, North America, northeastern United States as wild blueberries (lowbush) and British Columbia as cultivated blueberries (highbush). Blueberries contain phytochemicals, such as anthocyanins, which show potential antioxidant and anticancer effects. In some studies, the mechanism of anticancer activity has been reported, such as oxidative stress, products of oxidative stress such as increased apoptosis, DNA damage, inhibition of cell proliferation and production of proinflammatory molecules [43]. A study reported the chemopreventive efficacy and impact on angiogenesis and invasion of blueberries analysed by the ability to target PI3K/Akt, MAPK, NF-κB, and transforming growth factor β signalling in a hamster buccal pouch (HBP) carcinogenesis model. Administration of blueberry (200 mg/kg) inhibited the growth and progression of DMPA-induced OSCC by reducing the expression of the PI3K/Akt and TGF-β pathways. NF-κB activation is suppressed by preventing NF-κB p65 nuclear translocation, and in the MAPK pathway, no significant changes were observed [44]. In another study, blueberry and its main compound malvidin were reported to suppress STAT-3 phosphorylation in the SCC131 oral cancer cell line and induce mitochondrial-mediated apoptosis and cell cycle arrest in G1/S phase. Blueberry treatment of DMBA-painted hamsters at a 200 mg/kg concentration increased the tumour growth delay to 68.22% and reduced the multiplicity (0.87 ± 0.22) and tumour burden (26.27±2.82) [45]. The anticancer effects of pterostilbene (a polyphenolic compound found in blueberry fruit) on cisplatin-resistant human oral cancer (CAR) cells were reported in a recent study. pterostilbene treatment of CAR cells at concentrations of 25, 50, 70 and 100 µM (48 h) detected DNA breaks in cells, and an increase in the number of TUNEL (terminal deoxynucleotidyl transferase-mediated d-UTP nick end labelling)-positive cells was observed, indicating that pterostilbene mediates apoptosis of CAR cells through caspase-dependent signalling. Treatment of CAR cells with 50 and 75 µM pterostilbene (24 h) reduced the mRNA expression of multidrug resistance protein 1 (MDR1) and the phosphorylation of AKT at the Ser473 site [46].

### 4.4. Vaccinium macrocarpon *Aiton*

*Vaccinium macrocarpon* (cranberry), or the “wonder fruit”, is well known for its substantial therapeutic potential because of the availability of resveratrol and bioactive flavonoids, including anthocyanins, proanthocyanidins (PACs), and flavanols [47]. In another in vitro study, the antiproliferative effect of cranberry seed extract was analysed in the OSCC cell lines SCC25 and CAL27. Cranberry extract treatment of CAL27 cells upregulated key regulators of apoptosis, namely, caspase-8 (+181%) and caspase-2 (+327%), and a study of the relative change in proliferation between Days 1 and 3 showed that the GI_max_ value of 70 µg/mL cranberry extract reduced SCC25 proliferation by 36.3% in comparison to the baseline treatment control. Cranberry extract treatment of CAL25 cells upregulated the expression of c-myc (+29%), ODC (+371%), and p53 (+44%) [48]. In a recent in vitro study, the anticancer effect of *V. macrocarpon* hydroalcoholic fruit extract against the KB oral cancer cell line was examined, and the results showed that *V. macrocarpon* extract killed 50% of oral cancer cells, with an IC_50_ value of 3.564 µg/mL. A study reported that V. macrocarpon extract treatment of a normal fibroblast cell line (L929) resulted in a higher cell viability percentage and had an antiproliferative effect on the KB cell line [47].

### 4.5. Momordica charantia *L.*

*Momordica charantia* (bitter melon) contains bioactive compounds that show anticancer activities, such as phenolic acids, triterpene glycosides, triterpenoids, sterols, lectins, and flavonoids. Bitter melon is used in traditional folk medicine and cultivated in subtropical and tropical regions, including in India, tropical Africa, Indonesia, and China. Bitter melon extract and its components show anticancer activities by inhibiting the cell cycle, cancer stem cells, metastasis, and angiogenesis and enhancing reactive oxygen species generation [49]. In a recent study, the chemopreventive effect of bitter melon extract (BME) was analysed in HNSCC induced by the carcinogen 4-nitroquinoline 1-oxide (4-NQO). BME 30% *v*/*v* (600 mg/mouse) treatment reduced the occurrence of 4-NQO-induced carcinogenesis. BME treatment suppressed the expression of the immune checkpoint gene PDCD1/PD1 and the proinflammatory genes IL1b, IL23a, and s100a9, which were reported to have elevated expression during oral cancer development. BME treatment led to an 8.1-fold downregulation of MMP9 compared to that in 4-NQO-induced oral cancer tissues (6.8-fold upregulation of MMP9) [50]. In another study, the effects of BME on the metabolic pathways, lipid metabolism and glycolysis of human oral cancer cells were analysed. BME treatment of Cal27 and JHU022 oral cancer cell lines downregulated the protein and mRNA expression levels of glycolytic genes phosphofructo kinase (platelet) (PFKB), pyruvate kinase muscle (PKM), pyruvate dehydrogenase kinase 3 (PDK3), glucose transporter-1 (SLC2A1/GLUT-1) and lactate dehydrogenase alpha (LDHA). BME treatment also led to a significant reduction in the mRNA and protein levels of genes involved in fatty acid biogenesis, including the genes for ATP citrate lyase (ACLY), acetyl-CoA carboxylase 1 (ACC1), and fatty acid synthase (FASN). BME induced mitochondrial reactive oxygen species generation and CCAAT/enhancer-binding protein-homologous protein (CHOP) expression, which are associated with endoplasmic reticulum (ER) stress, and facilitated cell death via apoptosis in oral cancer [51].

### 4.6. Azadirachta indica *A. Juss*

**Figure 2:**
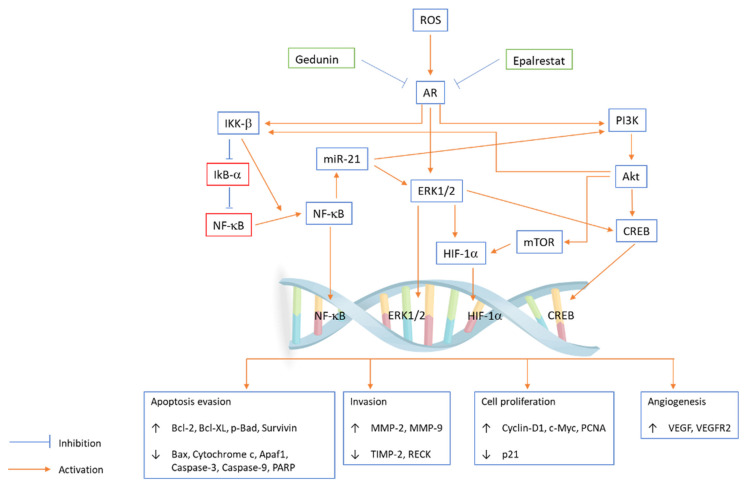
PI3K/Akt/mTOR/ERK/NF-κB signalling axis pathway plays an important role in regulating a broad range of cellular functions, including cell survival, growth, proliferation, invasion, apoptosis, angiogenesis, cell cycle and migration. Gedunin and epalrestat inhibit ROS generation and AR expression in the SCC131 cell line, and the coactivation of ERK and Akt is coupled with IKK/NF-κB signalling abrogation. Epalrestat and gedunin influence subcellular localization and phosphorylation, which modulate the expression of transcription factors and key oncogenic signalling kinases. Gedunin and epalrestat inhibit AR-mediated ROS signalling, which leads to the upregulation of Bcl-2, Bcl-XL, p-Bad, survivin, MMP-2, MMP-9, cyclin D1, c-Myc, PCNA, VEGF, and VEGFR2 and downregulation of Bax, cytochrome c, Apaf1, caspase-3, caspase-9, PARP, TIMP-2, RECK and p21.

*Azadirachta indica* (neem), belonging to the family Meliaceae, is a large perennial tree that is largely distributed in India. The neem tree is well known for its medicinal properties, such as insecticidal, antioxidant, antifungal, antitumour, and antibacterial properties [52]. Studies on different neem tree products, such as the limonoids nimbolide and azadirachtin and neem leaf glycoprotein, reported that they had anticarcinogenic properties against OSCC. In an in vitro study, the potential of gedunin alone or with epalrestat (AR inhibitor) to prevent hallmarks of cancer by inhibiting the downstream PI3K/Akt/mTOR/ERK/NF-κB signalling axis and aldose reductase (AR) in the oral cancer cell line SCC131 was examined. Gedunin and epalrestat treatment downregulated proangiogenic and proinvasive proteins in SCC131 cells and inhibited ROS generation and ARase expression (Figure 2). G1/S phase cell cycle arrest is associated with autophagy cell death following apoptosis [53]. Aqueous neem leaf extract is reported to have chemopreventive effects by regulating the enzymatic breakdown of glutathione in the oral mucosa [54].

### 4.7. Senegalia Catechu *(L. f.) P.J.H. Hurter & Mabb.*

*Senegalia catechu* (formerly known as *Acacia catechu*) is commonly known as black cutch, khair and babul and belongs to the Leguminosae family. It is native to East Africa and Asian countries, especially India. Bioactive compounds present in *S. catechu,* such as catechin, kaempferol, rutin, mesquitol, phenol, and aromadendrin, were isolated from the leaves, stems, roots, and bark and showed various biological activities. *S. catechu* shows various biological activities, including antifungal, vermifuge, astringent, anti-inflammatory, antimicrobial agent, antioxidant, chemopreventive activities, and are involved in oral health maintenance and wound healing. The cytotoxic and anticancer effects of *S. catechu* have been reported in COLO-205, HT-1080, and HeLa cell lines in vitro [55]. In a recent study, the ethanolic extract of *S. catechu* bark showed cytotoxic activity against the HSCC cell line SCC-25 with an IC_50_ value of 52.09 µg/mL. *S. catechu* extract treatment significantly upregulated the expression of apoptotic marker genes bcl-2, cytochrome c (Cyt-c), bax, and caspase-8. Ethidium bromide (EB)/acridine orange (AO) and propidium iodide (PI) staining showed nuclear membrane distortion and membrane blebbing, thus confirming that *S. catechu* extract induced apoptosis induction in SCC-25 cells. *S. catechu* extract at a 25 µg/mL concentration shows cell cycle arrest with 25% cell accumulation at S phase [56]. In another study, *S. catechu* seed ethanol extract was analysed for cytotoxic activity against the HSCC cell line SCC-25. Ethanolic seed extract treatment of the SCC-25 cell line caused cytotoxicity, with an IC_50_ value of 100 µg/mL. *S. catechu* seed extract treatment applied to SCC-25 cells resulted in downregulation of Bcl-2 gene expression and upregulation of apoptotic gene marker expression, including cytochrome c, Bax, caspase 8 and 9. Nuclear membrane distortion and membrane blebbing were observed based on PI and EB/AO staining, indicating that *A. catechu* induced apoptosis in SCC-25 cells [57].

### 4.8. Dracaena cinnabari *Balf.f.*

*Dracaena cinnabari* belongs to the Asparagaceae family and is commonly known as dragon’s blood tree, Damm Alakhwain (Yemen). It is native to the southern coast of Yemen and has been used since ancient times as a traditional folk medicine in different countries. Various biological activities of *D. cinnabari* have been reported, including anticancer, antifungal, antioxidant and cytotoxic activities [58]. In a recent in vitro study, *D. cinnabari* extract was analysed for its apoptosis-inducing and cytotoxic effects against the OSCC (H400) cell line, with an IC_50_ value of 5.9 µg/mL. Upregulation of caspase 9, caspase 8, caspase 3/7 activity and depolarization of mitochondrial membrane potential (MMP) were observed with *D. cinnabari* treatment. In the apoptotic protein array, the results showed that the Bcl-2 protein family regulates MMP by upregulating Bid, Bax, and Bad and downregulating Bcl-2. *D. cinnabari* extract treatment (48 and 47 h) increased the number (*p* < 0.05) of H400 cells in S phase, indicating apoptosis or cell cycle arrest [59]. In a recent in vivo study, the chemopreventive potential of a *D. cinnabari* resin methanol extract against a 4NQO-induced oral cancer animal model was observed. *D. cinnabari* extract treatment (1000 mg/kg) inhibited the expression of Bcl-2, p53, Ki-67 and cyclin D1 proteins and induced apoptosis by downregulating the Cox-2, Tp53, Bcl-2, cyclin D1, and epidermal growth factor receptor (EGFR) genes and upregulating the Casp3 and Bax genes [60]. In another study, the chemopreventive effect of *D. cinnabari* methanolic extract treatment on 4NQO-induced tongue SCC in rats was analysed. In an in vitro study, the *D. cinnabari* methanolic extract showed a cytotoxic effect on H103 cells, with an IC_50_ value of 5.5 µg/mL in a time- and dose-dependent manner. After treatment of H103 cells, morphological changes and cell migration reduction were observed, apoptosis was induced through the intrinsic (mitochondrial) pathway, and G2/M and S phase cell cycle arrest was observed. In an in vivo study, *D. cinnabari* methanolic extract-treated rats showed incidences of SCC of 500 mg/kg (28.6%), 100 mg/kg (57.1%) and 1000 mg/kg (14.3%) compared to 4NQO-induced cancer rats (85.7%) [61].

### 4.9. Piper nigrum *L.*

**Figure 3:**
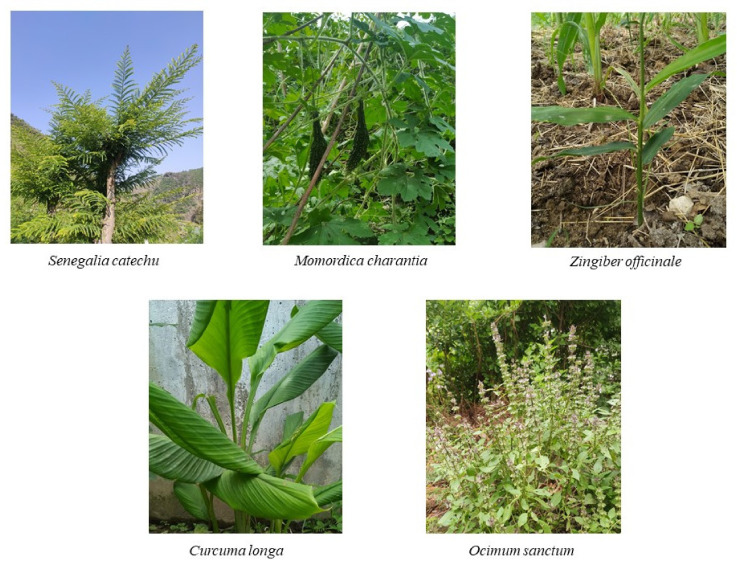
Medicinal plants used in oral cancer treatment.

*Piper nigrum* (black pepper), also known as ‘King of spices’, belongs to the Piperaceae family and is cultivated among tropical regions of India and Sri Lanka. It is known for its pungent flavour due to the presence of essential oils, volatile chemical compounds, and alkaloids (e.g., piperine) [62]. Black pepper is reported to have biological activities, such as anticancer, anti-larvicidal, pesticide, anti-Alzheimer’s, antidepressant, antiviral, and anti-inflammatory activities. A recent study reported that piperine presents cytotoxic activity against the human oral squamous carcinoma (HOSC) KB cell line [63]. In a recent in vivo study, the selective potential and cytotoxicity of the extracts of four *Piper* species, i.e., *Piper truncatum* (PT), *Piper arboretum* (PA), *Piper cernnum* (PC), and *Piper mollicomum* (PM), were analysed in OSCC cell lines (SCC9, SCC25, and SCC4). The three fractions PM (-L-D), PCa (-L-D) and PC (-L-D) (crude methanolic extract of leaves) with OSCC cell treatment showed toxicity, with IC_50_ values of 47.2, 94.2, and 47.5 µg/mL, respectively. An in vivo toxicology analysis of the PC-L-D fraction showed no significant alterations and less than 5% haemolysis [64]. Original photographs of a few important discussed plants are shown in Figure 3.

### 4.10. Zingiber Officinale *Roscoe*

*Zingiber officinale* (ginger) belongs to the Zingiberaceae family. It is cultivated mostly in Africa, India, South America, Nigeria, Thailand, and the Philippines and has been consumed worldwide as an herbal medicine and spice. Various bioactive compounds have been identified, such as terpene and phenolic compounds, gingerols, shogaols, and paradols, which have various bioactivities, including anticancer, antioxidant, antimicrobial, and anti-inflammatory activities [65]. In a recent in vitro study, zerumbone (a bioactive sesquiterpene found in Zingiber species) was analysed for its chemopreventive effect on OSCC cell lines. Zerumbone treatment of normal keratinocyte cells and OSCC cells shows cytotoxicity with IC_50_ values of 25 µM and 5 µM. Zerumbone inhibits the activation of the phosphatidylinositol-3-kinase-mammalian target of rapamycin (PI3K-mTOR) and chemokine C–X–C motif receptor 4 (CXCR4-RhoA) signalling pathways, leading to reduced OSCC cell viability. Zerumbone treatment (30 µM) inhibited invasion and migration, induced apoptosis in OSCC cells, and induced G_2_/M phase cell cycle arrest [66]. In another in vivo study, the cell proliferation and inflammatory effects of (6)-Shogaol ((6)-SHO) in DMBA-induced HBP carcinogenesis were examined by inhibiting the translocation of AP-1 and NF-κB. (6)-SHO treatment in DMBA-induced hamsters shows degradation of IκB-α, aberrant activation of AP-1, inhibition of nuclear factor kappa-B kinase subunit beta (IKKβ), c-jun, c-fos and NF-κB and upregulation of cell proliferative markers (PCNA, Ki-67 and cyclin D1) and inflammatory markers (interleukin-1 and -6, COX-2, TNF-α, inducible nitric oxide synthase (iNOS)). The results showed a reduction in the cell proliferative response and inflammation in DMBA-induced hamsters [67]. In another in vivo study, the chemopreventive effect of (6)-gingerol was analysed in DMBA-induced HBP carcinogenesis models. Oral supplementation with 20 mg/kg (6)-gingerol reduced the tumour incidence, tumour volume and tumour burden compared to DMBA treatment (1346.84 ± 81.19) and tumour volume (429.19 ± 28.29). (6)-Gingerol treatment induces HBP proapoptotic markers, inhibits cell proliferation markers: cyclin D1, inflammatory markers (interleukin [IL]-1β, TNF-α, cyclooxygenase-2, IL-6, inducible nitric oxide synthase) and proliferating cell nuclear antigen. Treatment with (6)-gingerol prevents HBP carcinogenesis by enhancing nuclear factor erythroid-2-related factor-2 expression, as DMBA-induced hamsters showed depleted Nrf2 signalling [68].

The medicinal plants and phytoextracts discussed in this review are demonstrated in Table 1.

## 5. Phytoextracts/Phytoconstituents for the Treatment of Oral Cancer

### 5.1. Curcumin

Curcumin is a nontoxic polyphenol compound found in *Curcuma longa* turmeric and is a well-known anticancer agent due to its effect on various biological pathways involved in oncogene expression, apoptosis, metastasis, tumorigenesis, and mutagenesis [39]. A study showed that curcumin has a stimulatory effect on the extrinsic apoptotic pathway activated by binding of Fas ligand and TNF-α “death activators” to their conforming cell surface receptors. It induces apoptosis in cancer cells at the G2 cell cycle phase by upregulating p53 gene expression [69]. A recent study reported that curcumin has an IC_50_ value of 10 µM, which reduces the progression and migration of TSCC cells (squamous cell carcinoma of the tongue), inhibits tumorigenesis, and promotes apoptosis [70]. Curcumin was found to be effective in suppressing cyclooxygenase 2 (*p* = 0.03) and NF-κB (*p* < 0.01) expression in experimentally induced oral squamous cell carcinoma [71].

### 5.2. Nimbolide

Nimbolide is a type of limonoid derived from *Azadirachta indica* (neem tree) that inhibits the proliferation of carcinogenic cells by inducing apoptosis. The aqueous extract of neem leaves regulates the enzymatic breakdown of glutathione and thereby exerts an anticarcinogenic effect in the oral mucosa [54]. In a recent study, the chemopreventive use of nimbolide against SCC4 and EAhy926 oral cancer cell lines was analysed. Nimbolide treatment upregulated reversion-inducing cysteine-rich protein with kazal motifs (RECK) by reducing hypoxia-inducible factor (HIF-1α), and miR-21 expression led to the downregulation of the chief mediators of angiogenesis and invasion. Nimbolide inhibits DMBA-induced hamster buccal pouch carcinomas that are closely related to human OSCC cells in gene expression signatures, metastasis, precancerous lesions and histology [72]. Nimbolide treatment activates apoptosis by inhibiting the cytoprotective autophagy shielding effect through modulation of the glycogen synthase kinase (GSK)-3β and Akt phosphorylation status in oral cancer cells SCC131 and SCC4 as well as in miR-126, ncRNAs, and homeobox transcript antisense intergenic RNA (HOTAIR) [73]. Supercritical CO2 neem leaf extract and its main bioactive compound nimbolide triggered the disruption of cell migration and signalling and efficiently reduced the levels of procancer inflammatory cytokines. The use of nimbolide and SCNE decreased cyclooxygenase-2 expression and NFkBp65 and downregulated pERK1/2, pSTAT3 and pAKT in SCC4 cells [74].

### 5.3. Resveratrol

Resveratrol is a type of natural phytoalexin produced from red wine, strawberries and grapes. It is reported to have antioxidant and in vivo and in vitro anticarcinogenic activity through mediating cell cycle arrest and various signalling pathways. Moreover, resveratrol induces cell apoptosis in oral cancer. A study on resveratrol suggested its use as a chemopreventive agent because the compound efficiently inhibited metastasis and invasion of the OSCC KB cell line in vitro [75]. A study reported that resveratrol shows an inhibitory effect on the growth of OSCC cells by enhancing the expression of cyclin A_2_, cyclin B_1_, and phospho-cdc2 (Tyr15) and the induction of apoptosis and cell cycle arrest in G_2_/M phase. The IC_50_ values of resveratrol after 48 h of treatment against the cell lines SCC-25, YD-38 and SCC-VII were 0.7, 1.0, and 0.5 μg/mL, respectively [76] Resveratrol can possibly decrease the metastasis and invasion of OSCC in oral cancer patients because it proficiently inhibits lysophosphatidic acid (LPA)-induced oral cancer cell invasion and epithelial-mesenchymal transition (EMT) by downregulating TWIST1 and SLUG (transcription factor) expression [77]. A recent study reported that Rab coupling protein (RCP) induces OSCC invasion and EMT by expressing Zeb1 and MT1-MMP. Resveratrol was reported to inhibit RCP-induced OSCC invasion by downregulating Zeb1 and β1 integrin expression [78].

### 5.4. Anthocyanin

Anthocyanins are natural polyphenolic pigments responsible for purple, red and blue colours in vegetables, fruits such as purple cabbage, grapes, and berries, and exhibit anticarcinogenic activity [79]. In a recent study, SCC4, SCC9, and SCC25 OSCC cell lines treated with anthocyanin showed morphological changes, including apoptotic cells, nuclear condensation, and fragmentation. Cell cycle arrest of SCC25 cells in the S-G_2_/M and G_0_/G_1_ stages with simultaneous upregulation of the sub-G_1_ fraction was observed using flow cytometry, which indicated cell death by apoptosis. Further involvement of caspase-3 activities in anthocyanin-induced apoptosis was verified using immunofluorescence analysis, which showed that in anthocyanin (IC_50_ and IC_80_ concentration)-treated SCC25 cells, the % caspase-3 expression level was between 1.5- and 3-fold (IC_50_—134%, IC_80_—267.5%) in comparison with control cells (89.6%) [80]. Furthermore, in a different study, anthocyanin showed a reduction in the viability of OSCC cells at a 250 µg/mL concentration at 48 h and inhibited the invasion and migration abilities of the OSCC cell lines SCC15 and Tca8113. Anthocyanin treatment in OSCC cells led to a significant increase in the protein expression of caspase-1, IL-1β, NLRP3 (nucleotide-binding oligomerization domain-like receptor pyrin domain-3) and was correlated with the activation of pyroptosis, and the administration of a caspase-1 inhibitor increased the invasiveness, cell viability, and migration compared to the anthocyanin-treated group [81].

### 5.5. Piperine

Piperine (1-piperidine) is an alkaloid found in the roots and fruits of *Piper longum* L. and *P. nigrum* L., and it is reported to have potential therapeutic activities, such as antioxidant, anticancer, immunomodulatory, and antimutagenic activities [82]. A study conducted to verify the chemoprevention efficacy of piperine during hamster buccal pouch carcinogenesis induced by DMBA found that tumour formation in hamster buccal pouches painted with DMBA alone was 100% after 14 weeks, the tumour burden was 1399.7 mm^3^ and the mean tumour volume was 378.3 mm^3^. Piperine oral administration completely prevents OSCC formation in hamster buccal pouches painted with DMBA (at a dose of 50 mg/kg body weight) alternately for 14 weeks. Piperine also restored the status of detoxifying agents, antioxidants, and lipid peroxidation in DMBA-painted hamsters [83]. A mechanism by which piperine induces apoptosis is suggested via the reduction in mitochondrial membrane potential (MMP) and liberation of ROS following cell cycle arrest and caspase-3 activation. The study results showed that the viability of the HOSC KB cell line was reduced significantly (*p* < 0.001) by treatment with various piperine concentrations (25 mM (90.14%), 50 mM (76.59%), 100 mM (52.39%), 200 mM (25.26%), and 300 mM (18.96%)), and the IC_50_ value of piperine in KB cells was 124 µM. A cell cycle study showed that piperine reduced the DNA content and arrested cells in the G2/M phase [63]. A recent study performed in silico docking simulations and showed that inside the ATP binding site in cyclin-dependent kinase 2, there is hydrogen bonding between residue Ser5 and piperine, which predicts protein-piperine hydrophobic interactions and antitumour molecular mechanisms. In vivo assays with supercritical fluid (SCF) inhibited the expression of the cell cycle proteins cyclin A and CDK2 and the antiapoptotic protein Bcl-xL and increased the expression of the proapoptotic proteins p53 and Bax [84]. In a recent study, the findings of molecular docking analysis showed an optimal hydrogen bonding length between the ligand piperine and cell cycle proteins, including cyclin D (2.62), cyclin T (1.5), CDK4 (3.26), and CDK2 (3.19) [85].

**Figure 4:**
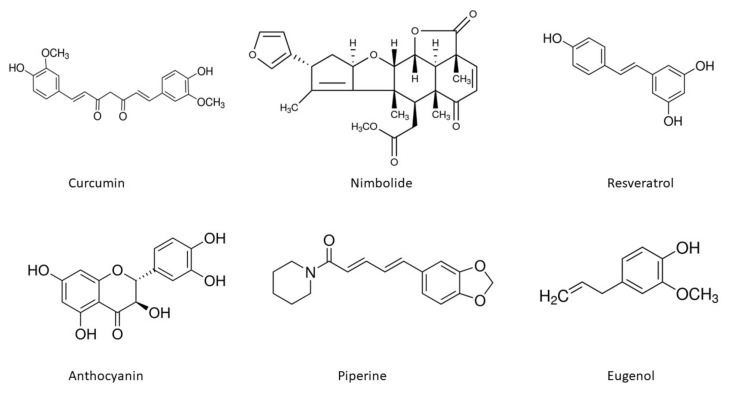
Chemical structures of phytoextracts useful in oral cancer treatment.

The chemical structures of the important phytoconstituents discussed in the review paper are shown in Figure 4.

### 5.6. Eugenol

Eugenol is a chief phytochemical present in clove oil extracted from cloves. Anticancer activities and eugenol-induced apoptosis molecular mechanisms have been reported against various cancers, such as leukaemia, melanoma, skin tumours, and osteosarcoma. However, a few studies have documented the oral cancer activities of eugenol. A study conducted to assess the mechanism of cytotoxicity induced by eugenol towards OSCC cells reported that eugenol induced nonapoptotic cell death in OSCC cells based on the metabolic profile. Eugenol treatment in OSCC cells induced oxidative stress by increasing the oxidized form of glutathione (69%) and the methionine sulfoxide/methione ratio (37%), increasing glycolytic metabolites and polyamines, and decreasing ATP utilization based on a 53% decrease in the ADP/ATP ratio and a 70% decrease in the AMP/ATP ratio [86]. In another study, the pathological and synergistic effects of oral mucositis were analysed using an N-succinyl chitosan gel delivery system of microemulsified sodium hyaluronate (0.2% *w*/*v*), eugenol (10% *v*/*v*), and honey (10% *v*/*v*). Compared with chitosan gel, N-succinyl chitosan mediated the efflux of eugenol and showed better results in rat buccal mucosal tissue ex vivo penetration studies, with an enhancement ratio of 1.71, and eugenol release from N-succinyl chitosan was 87.45 ± 0.14% in PBS buffer (pH-6.4) after 8 h in an in vitro study. N-succinyl chitosan orogel reduced inflammation in the oral mucosa of animals (*p* < 0.05) compared to the disease control [87]. In a recent in vitro study, the anticancer effect of eugenol extracted from *Cinnamomum verum* was analysed in an OSCC cell line. Eugenol treatment of OSCC 25 cells using the MTT cytotoxic assay showed an IC_50_ value of 24.71 µM, with concentrations ranging from 1.9 µg/mL to 1000 µg/mL. In a DNA fragmentation assay (48 h) of a 25 µM eugenol treatment of SCC cells, the agarose gel electrophoresis results showed a ladder pattern in eugenol-treated cells and the absence of a ladder pattern in untreated cells or negative controls, indicating apoptosis. A cell cycle analysis showed that a 25 µM eugenol treatment of the SSC 25 cell line resulted in a subsequent increase in the sub-G0 population and S phase arrest [88]. The roles of the phytoconstituents discussed in this review are demonstrated in Table 2.

## 6. Safety of Phytoextracts in Oral Cancer Treatment

In a recent phase I randomized clinical trial (RCT), the safety of curcuminoids (*Curcuma longa*) and an extract of *Bidens pilosa* L. as a mucoadhesive formulation (FITOPROT) for the treatment and prevention of oral mucositis induced by chemoradiotherapy in patients with HNSCC was investigated. A total of 20 healthy adult participants were divided into 2 groups: Group I received *B. pilosa* extract 20% *v*/*v* and curcuminoids 10 mg/mL (FITOPROT), and Group II received *B. pilosa* extract 40% *v*/*v* and curcuminoids 20 mg/mL (FITOPROT). The treatments were administered for ten consecutive days, three times daily. The results showed no change in micronuclei frequencies (*p* > 0.05) or cellular genotoxic effects (*p* > 0.05). In biochemical assays, no difference was found in pro45 inflammatory cytokine levels (*p* > 0.05) [89]. In another phase I CT, the effect of APG-157, a botanical drug containing curcumin and other polyphenols, on oral cancer was determined under the US Food and Drug Administration’s Botanical Drug Development division. In this study, 12 oral cancer patients and 13 normal controls were selected, and two transoral doses of 200 mg and 100 mg were given every hour for a 3 h period. After 3 h of treatment, reductions in IL-6, IL-8, and IL-1β levels and bacteroid species and peaks in circulating curcumin were observed in cancer subjects [90].

## 7. Antioxidants and Anticancer Activity Relationship

Antioxidants are compounds that inhibit oxidation, and they are commonly found in vegetables, medicinal plants, fruits (e.g., blueberries and cranberries) and grains. Antioxidants have a stable aromatic ring system that facilitates the delocalization of unpaired electrons in reactive oxygen species. These compounds eliminate the unpaired electron status of ROS by donating or accepting an electron to neutralize ROS and mitigate excessive ROS-induced oxidative stress by converting and scavenging ROS into less reactive species. A reduction in ROS levels can reverse the initiation of carcinogenesis [91]. Excess ROS can cause damage to RNA, DNA, and proteins, which further cause genetic alterations in cells, resulting in mutagenesis and eventually cancer. Low levels of ROS are essential in biological functions, including cell proliferation, growth, differentiation and cell survival. Studies have reported that ROS participate in both the p38 mitogen-activated protein kinase (p38MAPK) tumour suppressing pathway and Ras-Raf-MEK1/2-ERK1/2 oncogenic signalling. Some anticancer drugs cause cancer cell death by inducing ROS generation; for example, doxorubicin, an anthracycline-based anticancer drug, causes cancer cell death by accumulating hydroxyl radicals [92]. Some natural compounds also exhibit anticancer effects by inducing ROS; for example, curcumin treatment of OSCC cells induces apoptosis and induces intracellular ROS [41]. Gedunin and epalrestat treatment inhibits ROS generation, and piperine causes apoptosis by inducing ROS generation following cell cycle arrest [53]. Resveratrol induces the ROS-p38-p53 pathway by upregulating phosphorylated p38 MAPK gene expression [92]. The reason for cell death in cancer cells is due to the low tolerance thresholds of redox homeostasis; however, normal cells can tolerate drug-induced ROS stress [93]. Natural compounds show harmful or beneficial effects independent of their oxidative properties. Dietary antioxidants eliminate excess oxygen metabolites, which help reinforce our antioxidant system. Synergistic action between exogenous and endogenous antioxidants is often observed, which provides a balance between antioxidation protection and oxidant production and is believed to play an important role in maintaining a healthy biological system [94].

## 8. Conclusions and Future Perspectives

Since ancient times, plants and their bioactive substances have been used for medicinal purposes. Medicinal herbs are a gift from nature to humanity, and they have been demonstrated to possess significant anticancer properties. Phytochemicals found in some medicinal plant species have shown the ability to suppress the progression and development of oral cancer. Natural dietary phytoconstituents will continue to be a promising and active research topic in coming years. Further studies should be performed to explore promising phytochemicals from plants to assess their potential uses, mechanisms of action, pharmacokinetic profiles, metabolism, pharmacodynamic responses, toxicities, polymorphisms, drug-drug interactions, and dosage regimens for use as standard herbal medicines. Large-scale, well-controlled clinical trials are required to evaluate their efficacies, side effects, and safety. Furthermore, there is a need to further investigate the synergistic effects of phytochemical compound and chemotherapeutic drug combinations in cancer cells. Thus, the chemopreventive and anticancer properties of phytochemicals have attracted the interest of researchers in recent years because of their ability to provide anticancer treatment with low intrinsic toxicity and high effectiveness.

## Figures and Tables

**Table 1:** Role of medicinal plants in oral cancer.

Botanical Name	Study Type (*in vitro*/*in vivo*/ Clinical Trial) and Extract	Objective	Observation		Reference
*Ocimum sanctum* L. (Holy Basil)	Study—In vitro on KB mouth cell line Extract—Leaves aqueous extract	To analyse dose dependent cytotoxic activity of *O. sanctum* aqueous extract of leaves on oral cancer cell line	*O. sanctum* treatment show cytotoxic effect against oral cancer KB mouth cell with IC_50_ value of 10 µg/mL (aqueous extract of light leaves) and 20 µg/mL (aqueous extract of dark leaves) in 48 h MTT assay.		[36]
	Study—In vitro on HNSCC cell lines, HN4, HN30, HN12, HN31 Extract—Leaf ethanolic extract	To study the cytotoxic and anti-invasive effect of *O. sanctum* leaf ethanolic extract on HNSCC cell lines.	*O. sanctum* ethanolic extract (0.8 mg/mL) treatment shows decrease in cell viability of HNSCC cell lines HN30 (40%), HN31 (53%), HN4 (52%), and HN12 (40%).		[37]
			*O. sanctum* ethanolic extract with 0.4 mg/mL conc. inhibited MMP-2 activity in HN12, HN4 cells by 71% and 65% and MMP-9 activity in HN12, HN4 cells by 85% and 44%.		
			The invasion activity of HN12 and HN4 cells is inhibited by 30%.		
	Study—In vivo on DMBA induced OSCC hamsters Extract—Vicenin	To study anticancer effect of Vicenin-2 on DMBA-induced oral carcinogenesis in hamsters.	Vicenin-2 treatment (30 mg/kg) with DMBA-induced OSCC hamster improved antioxidant level, inhibited lipid peroxidation, and stopped tumor incidence.		[38]
			DMBA-induced hamsters with treatment of vicenin-2, halts the production of proinflammatory cytokines (TNF-α, IL-1β, IL-6) and inhibited immunohistochemical expression of cyclin-D1, Bcl-2, PCNA		
	Study—In vitro on OSCC cell line Ca9-22 Extract—Leaves (dry and aqueous)	To study the effect of dry leaves and aqueous extract of *O. sanctum* on OSCC cell line.	In 24 h MTT assay *O. sanctum* aqueous extract treatment, result shows HPC value 30 mg/L and MIC value of 5 mg/L and for dry extract HPC value is 35 mg/L and MIC value is 5mg/L.		[35]
*Curcuma longa* L. (Turmeric)	Study—In vitro on OSCC cell lines, H314, ORL-115. Extract—Curcumin		To access the correlation between intracellular copper levels and response to curcumin treatment in OSCC cell lines obtained from oral cancer patients.	Copper (250 µM) supplementation shows decrease in curcumin concentrations 25 μM to 5.3 μM at 48 h and 50 μM to 40.3 μM at 24 h, which inhibited 50% OSCC cell viability (IC_50_) 24 h MTT assay.	[41]
				Increase of copper level in OSCC cells treated with curcumin shows increase in Nrf2 level and significant induction of intracellular ROS. Combined treatment of curcumin with copper early apoptosis is observed.	
	Study—In vitro on OSCC cell lines, Ca9-22 and HSC-4. Extract—Curcumin		To study the effects of curcumin on HGF-induced EMT in OSCC cell lines.	Curcumin treatment in OSCC cells inhibited cell motility, HGF-induced EMT via c-Met blockade and reduced the expression of phosphorylated c-Met/ERK pathway which inhibit HGF-induced increase in vimentin level.	[42]
*Vaccinium corymbosum L. (Blueberry)*	Study—In vitro *and* in vivo on OSCC cell line SCC131 and DMBA painted hamster Extract—Malvidin, Blueberry powder	To access the potential of malvidin and blueberry to target STAT-3 (oncogenic transcription factor)	Blueberry and malvidin suppress STAT-3 phosphorylation in SCC131 oral cancer cell line and induced mitochondrial-mediated apoptosis and G1/S phase cell cycle arrest.		[45]
			Blueberry treatment to DMBA painted hamster with 200 mg/kg concentration, increased tumor growth delay to 68.22%, reduced the multiplicity (0.87±0.22)		
	Study—In vitro on CAR cell line CAL 27 Extract—Pterostilbene	To analyse anticancer effects of pterostilbene on cisplatin-resistant human oral cancer (CAR) cells.	Pterostilbene with 50, 75 µM concentration (24 h) treatment in CAR cells reduced the expression of MDR1, mRNA and phosphorylation of AKT on Ser473 site.		[46]
*Vaccinium macrocarpon Aiton (Cranberry)*	Study—In vitro on OSCC cell lines, SCC25, CAL27 Extract—Cranberry	To study anti-proliferative effect of cranberry against OSCC cell lines.	Cranberry extract treatment with CAL27 cells shows upregulation in key regulator of apoptosis, caspase-8 (+181%) and caspase-2 (+327%) and expression of c-myc (+29%), ODC (+371%), p53 (+44%).		[48]
			GI_max_ value of cranberry extract 70 µg/mL reduced SCC25 proliferation by 36.3% in comparison to baseline treatment control.		
	Study—In vitro on KB oral cancer cell line Extract—Fruit (hydroalcoholic)	*To examine the anticancer effect of V. macrocarpon hydroalcoholic fruit extract against KB oral cancer cell line is examined.*	*V. macrocarpon* extract kills 50% oral cancer cells with IC_50_ value of 3.564 µg/mL (24 h incubation, MTT cytotoxicity assay).		[47]
*Momordica charantia L.* *(Bitter melon)*	Study—In vivo on 4-NQO–induced cancer model Extract—Bitter melon extract	To access chemo preventive effect of bitter melon extract (BME) in HNSCC induced by 4-nitroquinoline 1-oxide (4-NQO) carcinogen	BME treatment (600 mg/mouse) suppress the expression of immune check point gene PDCD1/PD1, proinflammatory genes IL1b, IL23a, s100a9 and downregulate MMP9 pathway.		[50]
	Study—In vitro on human oral cancer cell line Cal27 and JHU022 Extract - Bitter melon extract	To analyse effect of Bitter melon extract on lipid metabolism and glycolysis pathways in human oral cancer cells.	BME treatment on oral cancer cell lines shows downregulation in protein and mRNA expression levels of PFKB, PKM, PDK3, SLC2A1/GLUT-1 and LDHA, induced mitochondrial ROS generation and CHOP expression associated with endoplasmic reticulum (ER)-stress, facilitated cell death via apoptosis in oral cancer.		[51]
*Azadirachta indica* A. Juss. (Neem tree)	Study—In vitro on oral cancer cell line SCC131 Extract—Gedunin	To study the potential of gedunin alone or with epalrestat to prevent hallmarks of cancer by inhibiting downstream PI3K/Akt/mTOR/ERK/NF-κB signalling axis and ARase in oral cancer cells.	Gedunin and epalrestat treatment shows, downregulation of proangiogenic and pro-invasive proteins in SCC131 cells and inhibited ROS generation, ARase expression. G1/S phase cell cycle arrest is associated with autophagy cell death following apoptosis		[53]
*Senegalia catechu* (L.f.) P.J.H. Hurter & Mabb. (Black cutch)	Study—In vitro on HSCC cell line (SCC-25) Extract—Bark ethanolic extract	To analyse cytotoxic activity of ethanolic extract of *S. catechu* bark against HSCC cells.	Ethanolic extract of *S. catechu* bark shows cytotoxic activity in MTT assay (24 h) against HSCC cell line (SCC-25) with IC_50_ value of 52.09 µg/mL and 25 µg/mL concentration shows cell cycle arrest with 25% cell accumulation at S phase.		[56]
	Study—In vitro on HSCC cell line (SCC-25) Extract—Seed ethanolic extract	To analyse cytotoxic activity of ethanolic extract of *S. catechu* seed against HSCC cells.	Ethanolic seed extract treatment with SCC-25 cell line caused cytotoxicity (24 h, MTT assay) with IC_50_ value of 100 µg/mL and shows downregulation in Bcl-2 gene expression and upregulation in apoptotic gene marker expressions including cytochrome c, Bax, caspase 8 and 9.		[57]
*Dracaena cinnabari* Balf.f.(Dragon blood tree)	Study—In vitro on OSCC cell line -H400 Extract—Resin methanolic extract		To evaluate the apoptosis-inducing and cytotoxic effects of *D. cinnabari* on OSCC cells.	*D. cinnabari* treatment shows cytotoxic effect (MTT assay, 72 h) against OSCC (H400) cell line with IC_50_ value of 5.9 µg/mL, increase in caspase 8, caspase 9, caspase 3/7 activity, depolarization of mitochondrial membrane potential (MMP) and cell cycle arrest at S phase.	[59]
	Study—In vivo on 4-NQO–induced cancer model Extract—Resin methanolic extract		To study the chemo preventive efficacy of *D. cinnabari* on a 4NQO-induced oral cancer animal model.	*D. cinnabari* extract treatment with 1000 mg/kg inhibits expression of Ki-67, Bcl-2, p53 and cyclin D1 proteins and induced apoptosis by downregulation of Bcl-2, Cox-2, Tp53, upregulation of Casp3 and Bax genes.	[60]
	Study—In vivo on 4NQO-induced tongue carcinogenesis and in vitro *on* tongue squamous cell carcinoma cell line (H103) Extract—Resin methanolic extract		To study the chemo preventive activity of *D. cinnabari* against 4NQO-induced tongue carcinogenesis in rat and apoptosis induction of *D. cinnabari* on tongue squamous cell carcinoma cell line.	*D. cinnabari* methanolic extract treated rats shows incidence of SCC 100 mg/kg (57.1%), 500 mg/kg (28.6%) and 1000 mg/kg (14.3%) as compared to 4NQO induced cancer rats 85.7%.	[61]
				Cytotoxic effect (72 h, MTT assay) on H103 cells with IC_50_ value of 5.5 µg/mL in time and dose dependent manner, apoptosis induction is through intrinsic (mitochondrial) pathway and G2/M and S phase cell cycle arrest is observed.	
*Piper nigrum* L. (Black pepper)	Study—In vitro on OSCC cell line: SCC9, SCC25, SCC4 Extract- Leaves	To analyze cytotoxicity of four Piper species extract, *Piper truncatum* (PT), *Piper arboretum* (PA), *Piper cernnum* (PC), *Piper mollicomum* (PM).	In MTT assay, 48 h treatment cytotoxicity against OSCC cells with IC_50_ value of PC (-L-D) 47.2 µg/mL, PM (-L-D 94.2 µg/mL, PCa (-L-D) 47.5 µg/mL is observed.		[64]
*Zingiber officinale* Roscoe (Ginger)	Study—In vitro on OSCC cell line ORL-48 and ORL-115 Extract—Zerumbone		To examine OSCC cells were sensitive to zerumbone treatment and find the molecular pathways involved in the mechanism of action.	Zerumbone treatment on normal keratinocyte cells and OSCC cells shows cytotoxicity (MTT assay, 72 h) with IC_50_ value of 25 µM and 5 µM, inhibit activation of PI3K-mTOR and CXCR4-RhoA signalling pathway and induced apoptosis in OSCC cells, and shows cell cycle arrest at G_2_/M phase.	[66]
	Study—In vivo on DMBA induced hamster buccal pouch carcinogenesis Extract - Shogaol		To study the effect of (6)-Shogaol on cell proliferation and inflammation by inhibiting the translocation of AP-1 and NF-κB in DMBA induced HBP carcinogenesis	(6)-Shogaol treatment in DMBA induced hamsters shows degradation in IκB-α, aberrant activation of AP-1, IKKβ, c-jun, c-fos and NF-κB and upregulation of cell proliferative markers (PCNA, Ki-67 and Cyclin-D1), inflammatory markers (interleukin-1 and -6, COX-2, TNF-α, iNOS.	[67]
	Study—In vivo on DMBA induced hamster buccal pouch carcinogenesis Extract—Gingerol		To examine the chemo preventive effect of (6)-gingerol on DMBA induced hamster buccal pouch carcinogenesis models.	Oral supplementation of (6)-gingerol 20 mg/kg shows reduction in tumor incidence, tumor volume and tumor burden as compared to DMBA treatment tumor burden (1346.84 ± 81.19), tumor volume (429.19 ± 28.29).	[68]
				(6)-Gingerol treatment prevent HBP carcinogenesis, by enhanced nuclear factor erythroid-2- related factor-2 (Nrf2) expression as DMBA induced hamster shows depletion of Nrf2 signalling.	

**Table 2:** Role of phytoextract in oral cancer.

Phyto-Extract	Source	Study Type (*in vitro*/*in vivo*/ Clinical Trial)	Objective	Observation	Reference
Curcumin	*Curcuma longa* L.	Study—In vitro on HNSCC cells	To study therapeutic activity of curcumin against HNSCC cells.	Curcumin shows stimulatory effect on extrinsic apoptotic pathway activated by binding of Fas ligand and TNF-α to their conforming cell surface receptors. It induces apoptosis in cancer cells at the G2 cell cycle phase by upregulation of p53 gene expression.	[69]
		Study—In vitro study on TSCC cell line	To study anticancer potential of curcumin against TSCC cells.	Curcumin with IC_50_ value of 10 µM (MTT assay, 24 h) reduces the progression and migration of TSCC cells, inhibits tumorigenesis, and promotes apoptosis.	[70]
		Study—In vivo on DMBA-induced OSCC in Sprague Dawley rats	To evaluate the anti-cancer potential of curcumin on OSCC based on the expression of cyclooxygenase 2 and nuclear factor kappa B during epithelial dysplasia stage.	Curcumin treatment found to be effective in suppressing cyclooxygenase 2 (*p* = 0.03) and nuclear factor kappa B (NFKB) (*p* < 0.01).	[71]
Nimbolide	*Azadirachta indica*	Study—In vitro study on OSCC cell line SCC4 and in vivo *on* DMBA-induced HBP carcinogenesis.	To study the chemotherapeutic effect of nimbolide based on modulation of the expression of key molecules involved in angiogenesis, invasion, and the upregulation of RECK.	Nimbolide treatment upregulates—RECK by reducing miR-21 and HIF-1α expression leads to downregulation of MMP activity, blockade of notch signalling and VEGF. Nimbolide significantly inhibits DMBA-induced HBP carcinomas.	[72]
		Study—In vitro study on OSCC cell lines SCC131 and SCC4.	To analyze the effect of nimbolide on autophagy and the time point at which the phosphorylation status of PI3K, GSK-3β determine the choice between apoptosis and autophagy in oral cancer cells.	Nimbolide treatment activates apoptosis by inhibiting shielding effects of cytoprotective autophagy through modulation in the phosphorylation status of GSK-3β and Akt in oral cancer cells SCC131 and SCC4, as well as in miR-126, ncRNAs, and HOTAIR.	[73]
Super critical CO_2_ Neem leaf extract (SCNE)		Study—In vitro study on OSCC cell line SCC4, Cal27, and HSC3.	To study anticancer effects of SCNE and nimbolide against OSCC cell lines while inflammation, migration and proliferation were analyzed over time.	Nimbolide treatment caused disruption of cell migration, cell signaling and efficiently reduced pro-cancer inflammatory cytokines. Use of nimbolide and SCNE decreased NFkBp65, COX2 expression, and downregulated pAKT, pERK1/2, and pSTAT3 in SCC4 cells.	[74]
Resveratrol	*Vaccinium corymbosum*	Study—In vitro on OSCC cell lines, SCC-VII, SCC-25, and YD-38.	To examine the chemo preventive effect of resveratrol against oral squamous cancer cell lines,	Resveratrol shows an inhibitory effect on the growth of OSCC oral cancer cells by enhanced expression of cyclin A_2,_ cyclin B_1,_ and phosphor-cdc2 (Tyr 15) and the induction of G_2_/M phase cell cycle arrest and apoptosis.	[76]
				IC_50_ value of resveratrol (48-h treatment, MTT assay) against SCC-25, YD-38 and SCC-VII cell lines were found to be 0.7, 1.0, and 0.5 μg/mL.	
		Study—In vitro on OSCC cell line YD-10B.	To analyze the potential therapeutic efficiency of resveratrol in oral cancer patients.	Resveratrol treatment decrease metastasis and invasion of OSCC cells, as it proficiently inhibited LPA- induced oral cancer cell invasion and EMT by downregulating TWIST1 and SLUG expression.	[77]
		Study—In vitro on OSCC cell line YD-9, YD-38 and YD-10B.	To study chemo preventive effect of resveratrol on RCP-induced OSCC.	Resveratrol treatment inhibited the RCP—induced OSCC invasion by Zeb1 and β1 integrin expression downregulation.	[78]]
Anthocyanin	*Bridelia retusa*	Study—In vitro on OSCC cell line SCC4, SCC9 and SCC25	To study anti-metastatic potential of anthocyanin against oral squamous carcinoma cells.	Cell cycle arrest of SCC25 cells in S-G_2_/M and G_0_/G_1_ stages with up-regulation of the sub-G_1_ fraction is observed, indicate cell death by apoptosis. Anthocyanin treatment in SCC25 cells (MTS assay, 42 h), reported caspase-3 expression level between 1.5—3 folds (IC_50_—134%, IC_80_—267.5%) in comparison with control cells (89.6%).	[80]
	*Vaccinium corymbosum*	Study—In vitro on OSCC cell lines SCC15, HaCaT and Tca8113	To examine the potential inhibitory effects of anthocyanin on OSCC and find effective targets for therapy.	Anthocyanin shows, reduction in viability of OSCC cells with 250 µg/mL of concentration (CCK8 assay, 48 h). Anthocyanin treatment in OSCC cells shows a significant increase in protein expression of caspase-1, IL-1β, NLRP3.	[81]
Piperine	*Piper longum*	Study- In vivo *on* DMBA induced HBP carcinogenesis	To study the chemo-preventive efficacy piperine against (DMBA)-induced hamster buccal pouch carcinogenesis.	Piperine treatment on DMBA induced HBP carcinoma, prevents OSCC formation in DMBA—painted hamsters with a dose of 50 mg/kg body weight, given alternate days to DMBA painting for 14 weeks completely. Piperine restored the status of detoxifying agents, antioxidants, and lipid peroxidation in DMBA- painted hamsters	[83]
		Study—In vitro on OSCC KB cell line	To study the anti-cancer potential of piperine on human OSCC cells.	Piperine induce apoptosis via the reduction in MMP and ROS liberation following caspase-3 activation and cell cycle arrest. Cell cycle study showed that piperine reduced the DNA content and arrest cells in the G2/M phase.	[63]
		Study- In silico on CDK-2 and Bcl-xL	To analyze the anti-tumor activity of piperine rich extract by SFE from black pepper.	In silico docking simulations reported, inside the ATP binding site in CDK2 there is hydrogen bonding between residue Ser5 and protein.	[84]
Eugenol		Study—In vitro on human OSCC cell line HSC-2	To study the effect of eugenol treatment on the metabolic profiles of a human OSCC cell line.	Eugenol treatment in OSCC cells, induce oxidative stress by increase in oxidized form of glutathione (69%) and methionine sulfoxide/methione ratio (37%), increase in glycolytic metabolites and polyamines, decline of ATP utilization by 53% decrease in ADP/ATP ratio and 70% decrease in AMP/ATP ratio.	[86]
		Study—In vitro *and* Ex vivo on rat buccal mucosal tissue.	To study N-succinyl chitosan gel delivery system of micro emulsified honey, sodium hyaluronate and eugenol for synergistic effects on various pathological factors of oral mucositis.	N-succinyl chitosan orogel shows inflammation reduction in oral mucosa of animals (*p* < 0.05) compared to disease control. Eugenol release from N-succinyl chitosan were 87.45±0.14% in PBS buffer (pH-6.4) after 8 h.	[87]
	*Cinnamomum verum*	Study—In vitro on OSCC cell line SCC25.	To study the anticancer effects of eugenol against OSCC cells.	Eugenol treatment on OSCC cells using MTT cytotoxic assay (72 h) shows IC_50_ value of 24.71 µM with concentration ranging between 1.9 µg/mL and 1000 µg/mL. In cell cycle analysis, 25 µM eugenol treatment with SSC 25 cell line shows subsequent increase in sub-G0 population and cell cycle arrest at S Phase.	[88]
